# Gender and ethnic differences in the control of hyperlipidaemia and other vascular risk factors: insights from the CEntralised Pan-South African survey on tHE Under-treatment of hypercholeSterolaemia (CEPHEUS SA) study

**DOI:** 10.5830/CVJA-2013-043

**Published:** 2013-08

**Authors:** Naomi Rapeport, Colin Leslie Schamroth, Jacobus Blom Dirk

**Affiliations:** Milpark Hospital, Johannesburg, South Africa; Milpark Hospital, Johannesburg, South Africa; Division of Lipidology, Department of Medicine, University of Cape Town, South Africa

## Abstract

**Aim:**

The aim of the CEntralised Pan-South African survey on tHE Under-treatment of hypercholeSterolaemia (CEPHEUS SA) was to evaluate the current use and efficacy of lipid-lowering drugs (LLDs) in urban patients of different ethnicity with hyperlipidaemia, and to identify possible patient characteristics associated with failure to achieve low-density lipoprotein cholesterol (LDL-C) targets. There is little published data on LDL-C attainment from developing countries.

**Methods:**

The survey was conducted in 69 study centres in South Africa and recruited consecutive patients who had been prescribed LLDs for at least three months with no dose adjustment for six weeks. All patients provided written consent. One visit was scheduled for data collection, including fasting lipid and glucose, and HbA_1c_ levels.

**Results:**

Of the 3 001 patients recruited, 2 996 were included in the final analyses; 1 385 subjects were of Caucasian origin (818 male), 510 of African ancestry (168 male), 481 of mixed ancestry (222 male) and 620 of Asian origin (364 male). Only 60.5% of patients on LLDs for at least three months achieved the LDL-C targets recommended by the NCEP ATP III/2004 updated NCEP ATP III guidelines and 52.3% the fourth JETF/South African guidelines. African females were on average younger than females of other ethnic origins, and had the lowest smoking rates but the highest prevalence of obesity, hypertension, the metabolic syndrome and diabetes mellitus (DM), with the worst glycaemic control. Although women were less likely than men to reach goal [OR 0.65 (CI 0.54–0.77), *p* < 0.001 for NCEP ATP III guidelines and OR 0.76 (CI 0.64–0.91), *p* < 0.003 for fourth JETF guidelines], women of African ancestry were just as likely not to reach goal as their Caucasian counterparts.

**Conclusion:**

The results of this survey highlight the sub-optimal lipid control achieved in many South African patients, and profile important gender and ethnic differences. Control of cardiovascular disease risk factors across gender and ethnic groups remains poor.

## Abstract

Elevated serum lipid levels have been identified as one of the modifiable risk factors in the aetiology of cardiovascular disease (CVD). The INTERHEART study established that elevated lipid levels was the greatest contributor to the development of myocardial infarction worldwide.^1^ Multiple studies have evaluated the control of serum lipid levels in clinical practice but these studies originate almost exclusively from the developed nations of North America and Europe.^2^-^8^ Cardiovascular risk-factor control is poorly studied in developing nations, and in particular, knowledge of the control of lipid levels is largely unknown. In the limited published data, there is no specific information on gender and/or ethnic differences.^9^

Different authorities, such as the Joint European Task Force (JETF) and the United States National Cholesterol Educational Program Adult Treatment Panel III (NCEP ATP III) have developed clinical guidelines for the management of CVD risk, and there are extensive data showing that modification of risk factors can delay the development of CVD or prevent recurrent events in those with CVD at baseline.^10^-^13^ Over time, these guidelines have proposed progressively lower targets for CVD risk factors on the basis of evidence from clinical studies demonstrating that cardiovascular risk is further reduced by more rigorous risk-factor control. Aggressive low-density lipoprotein cholesterol (LDL-C) lowering remains the cornerstone of lipid management.

The CEntralised Pan-South African survey on tHE Undertreatment of hypercholeSterolaemia (CEPHEUS SA) was initiated to detect and quantify the degree of undertreatment of hypercholesterolaemia in South Africa, and the full overall results have been published.^14^ This study afforded us an opportunity to study both gender and ethnic differences in the prevalence of these CVD risk factors in a developing world population on drug treatment for elevated serum lipid levels, and forms the basis of this article.

## Methods

CEPHEUS was a non-interventional study conducted in South Africa between November 2009 and April 2010. To be representative of the varied population demographics in the country, 101 investigators in 69 urban study centres were involved in recruiting patients; 67 were general/primary healthcare practitioners, 13 were cardiologists, seven endocrinologists, and 14 internists/specialist physicians. Investigators were drawn from both the public and private sectors. Public sector sites were located in tertiary level referral hospitals.

Subjects 18 years or older who had been receiving lipidlowering drugs (LLDs) for at least three months (without dose adjustments for at least six weeks) were eligible. Consecutive patients who came for their regular scheduled visit to the doctor/clinic were invited to participate in the survey. Patients who agreed to participate provided informed written consent.

CEPHEUS was a single-visit non-interventional study. Each patient’s record form documented patient demographics, current LLD treatment, smoking status, known diabetes mellitus (DM), family history of premature vascular disease, known arterial hypertension (HT) and cardiovascular medical history. Physical examination by the investigator was limited to measurement of height, weight, waist circumference and blood pressure. A fasting blood sample was drawn to evaluate the serum lipid profile [total cholesterol, LDL-C, high-density lipoprotein cholesterol (HDL-C), triglycerides and apolipoprotein (apo) AI and apo B], fasting blood glucose (FG) and glycosylated haemoglobin (HbA_1c_) levels.

The primary endpoint was the percentage of patients who achieved the LDL-C goals according to either the NCEP ATP III/2004 updated NCEP ATP III guidelines or the fourth JETF/South African guidelines, which were current in South Africa at the time the CEPHEUS SA study was conducted. Secondary endpoints included achievement of LDL-C goals in patients with and without features of the metabolic syndrome, and primary versus secondary prevention.

The parent study (CEPHEUS-Europe) has been registered with the US National Institutes of Health (ClinicalTrials.gov), number NCT00542867. The CEPHEUS study was sponsored by AstraZeneca. The sponsor oversaw data collection and monitored study sites. The authors had full access to the study database and all analyses reported here were performed independently of the sponsor.

## Statistical analysis

We subdivided the cohort by gender and ethnicity for the purposes of this analysis. The four major ethnic groups in South Africa were black Africans, Caucasians, Asians (including patients of Indian descent) and patients with mixed ancestry. The risk category was determined for each patient and we calculated a dichotomous variable for each patient indicating whether their LDL-C had reached the guideline mandated target level.

We generated descriptive statistics for all clinical and laboratory parameters, following subdivision by gender only, and then following subdivision by both ethnicity and gender. We analysed the effect of ethnicity and gender on goal attainment using logistic regression with the logit function in a model that incorporated ethnicity and gender simultaneously.

We calculated odds ratios and 95% confidence intervals for the probability of not attaining LDL-C goal. The probability of not attaining LDL-C goal was referenced against Caucasian ethnicity and male gender, for which the odds ratio was set as 1. All *p*-values are two-sided and we regarded *p* < 0.05 as statistically significant. All analyses were performed with Statistica [StatSoft Inc (2011), STATISTICA (data analysis software system) version 10, www.statsoft.com].

## Results

A total of 3 001 patients consented to participate in the survey. Full data sets were available from 2 996 patients and form the basis of this report. About two-thirds of patients were recruited from the private healthcare sector, with the remaining one-third coming from public sector institutions.

Demographic, anthropometric and clinical data are shown in Table 1. Of the total group, 47.1% had known DM but 2.4% of patients who did not give a history of DM had FG levels that would qualify for the diagnosis of DM. Glycaemic control in patients who gave a history of DM was generally poor, with a mean HbA_1c_ level of 8.33%.

**Table 1:** Baseline Characteristics

*Characteristics*	*Entire study*		*Caucasian*		*African*		*Mixed ancestry*		*Asian*	
	*Male*	*Female*	*Male*	*Female*	*Male*	*Female*	*Male*	*Female*	*Male*	*Female*
Number	1572	1424	818	567	168	342	222	259	364	256
Mean age (years)	59.2	59.6	60.9	62.0	57.4	57.4	58.1	59.0	56.8	58.0
Current smoker (%)	18.6	10.8	15.9	14.3	15.6	2.6	25.1	19.7	21.4	5.1
Family history of vascular disease (%)	27.0	29.5	29.0	36.3	4.1	11.1	25.2	30.9	39.2	37.5
Mean body mass index (kg/m^2^)	29.2	30.8	30.0	29.4	29.3	34.2	29.0	31.2	27.6	29.4
Mean waist circumference (cm)	101.0	101.0	105.7	95.6	101.5	102.2	101.7	100.4	99.4	97.6
Known diabetes mellitus (%)	45.8	48.5	34.4	26.8	70.6	74.9	54.1	54.5	38.7	55.1
Known systemic hypertension (%)	68.8	74.6	64.7	64.7	84.6	88.9	76.6	84.2	65.8	67.9
History of coronary heart disease (%)	45.8	23.9	46.3	19.6	19.0	14.3	58.1	38.2	49.4	31.6
History of cerebrovascular disease (%)	5.8	4.8	5.3	4.4	5.9	6.1	6.3	4.2	6.3	4.2
History of peripheral arterial disease (%)	6.2	3.5	7.8	3.2	3.0	2.6	6.8	6.2	3.3	2.7

The prevalence of a history of HT in this study was 71.6%. The mean systolic blood pressure in the entire study cohort was 133.2 mmHg, with a diastolic pressure of 80.2 mmHg. In those subjects with a history of HT (Table 2), the mean systolic blood pressure was 136.1 mmHg, with a diastolic pressure of 81.3 mmHg. African-ancestry males had the highest systolic blood pressure and females of mixed ancestry the highest diastolic blood pressure but the inter-ethnic differences were small.

**Table 2:** Control Of Diabetes And Hypertension

*Condition*	*Entire study*		*Caucasian*		*African*		*Mixed ancestry*		*Asian*	
	*Male*	*Female*	*Male*	*Female*	*Male*	*Female*	*Male*	*Female*	*Male*	*Female*
Diabetic patients										
*Number*	*718*	*690*	*279*	*152*	*118*	*256*	*120*	*139*	*200*	*141*
*Glucose (mmol/l)*	*7.93*	*8.44*	*7.46*	*8.08*	*8.12*	*9.12*	*8.17*	*7.86*	*8.24*	*8.19*
*HbA_1c_ (%)*	*7.94*	*8.73*	*7.33*	*7.64*	*8.66*	*9.60*	*8.19*	*8.52*	*8.26*	*8.57*
Hypertensive patients										
Number	1081	1063	529	367	142	304	170	218	240	174
Systolic blood pressure (mmHg)	134.9	137.3	135.6	135.6	137.3	137.1	136.6	141.9	130.6	135.3
Diastolic blood pressure (mmHg)	81.7	80.8	82.2	79.3	83.8	81.5	81.3	81.2	79.8	80.0

More Caucasian patients were receiving LLDs for primary prevention compared to those of African ancestry, few of whom were on treatment for primary prevention. The majority were receiving treatment for the CVD risk equivalent of DM.

The percentage of African patients who were on LLDs for coronary artery disease (CAD) was lower than that seen in the other ethnic groups. The prevalence of CAD was higher in males than in females in all ethnic groups. Among male participants, CAD rates were highest in men of Caucasian, Asian and mixed ancestry. Among the women, the highest prevalence of CAD was in women of mixed and Asian ancestry. Few African patients gave a family history of CAD and the percentage of smokers was also lowest among the African patients. Most patients (95.9%) were on LLD monotherapy, with this being almost exclusively (98.9%) statin based.

The on-treatment lipid and FG values are listed in Table 3. Overall, patients of African ancestry had lower TC, LDL-C, and non-HDL-C levels and higher FG levels than subjects of other ethnic groups. In the cohort with DM (Table 2), the Africanancestry patients had the highest levels of HbA1c, both for males and females.

**Table 3:** Laboratory Results

*Laboratory parameters*	*Entire study*		*Caucasian*		*African*		*Mixed ancestry*		*Asian*	
	*Male*	*Female*	*Male*	*Female*	*Male*	*Female*	*Male*	*Female*	*Male*	*Female*
Total cholesterol (mmol/l)	4.72	5.06	4.75	5.26	4.44	4.57	4.78	5.20	4.75	5.13
LDL cholesterol (mmol/l)	2.63	2.85	2.62	2.93	2.44	2.55	2.65	3.01	2.72	2.91
HDL cholesterol (mmol/l)	1.21	1.41	1.25	1.53	1.19	1.31	1.15	1.36	1.19	1.33
Triglycerides (mmol/l)	2.01	1.79	2.03	1.80	1.83	1.55	2.31	1.83	1.88	1.99
Non-HDL cholesterol (mmol/l)	3.51	3.65	3.51	3.73	3.25	3.26	3.63	3.84	3.56	3.80
Glucose (mmol/l)	6.52	6.76	6.0	5.8	7.2	8.1	6.8	6.7	7.1	7.0
Glycosylated haemoglobin (%)		7.34	638	6.31		8.74		7.48		7.91

The primary outcome or percentage of patients reaching LDL-C targets is given in Table 4. Overall 60.5% of patients reached goal as per the NCEP ATP II guidelines and 52.3% according to the JETF guidelines. Differences in attainment of goal were noted. Patients of mixed ancestry were less likely to get to either of the two goals, with the exception of mixed-ancestry males, who had similar not-at-goal percentages as the male patients of Asian ancestry. Females subjects were less likely to get to goal, both for the NCEP ATP III [OR 0.65 (CI 0.54–0.77), *p* < 0.001] and JETF [OR 0.76 (CI 0.64–0.91), *p* < 0.003] guidelines. This difference was maintained across the various ethnic groups.

**Table 4:** Attainment Of Primary Goal (%)

*Guidelines*	*Entire study*		*Caucasian*		*African*		*Mixed ancestry*		*Asian*	
	*Male*	*Female*	*Male*	*Female*	*Male*	*Female*	*Male*	*Female*	*Male*	*Female*
NCEP ATP III	63.4	56.8	67.3	61.5	68.4	60.8	61.0	41.7	58.7	52.7
EAS/ESC	55.0	49.3	57.5	50.1	62.3	55.8	44.3	42.0	51.6	48.0

The secondary outcomes or percentages of patients receiving LLDs with the metabolic syndrome, and the breakdown of those receiving LLDs for primary versus secondary prevention is given in Table 5.

**Table 5:** Secondary Outcome Variables

*Variables*	*Entire study*		*Caucasian*		*African*		*Mixed ancestry*		*Asian*	
	*Male*	*Female*	*Male*	*Female*	*Male*	*Female*	*Male*	*Female*	*Male*	*Female*
Metabolic syndrome (%)	65.2	71.5	62.8	55.7	72.6	88.0	71.2	77.6	63.7	78.1
Primary prevention (%)	40.3	48.7	47.4	66.7	32.7	24.9	32.4	44.0	34.1	45.7
Secondary prevention (including DM) (%)	59.7	51.3	52.6	33.3	67.3	75.1	67.6	56.0	65.9	54.3

## Discussion

The World Health Organisation has indicated that cardiovascular disease will be the number one cause of mortality in the developing world by 2020.^15^ Subjects with cardiovascular disease in underdeveloped countries tend to exhibit mortality 10 or more years before their counterparts from the developed nations.^16^ These factors indicate that it is imperative to address all cardiovascular risk factors aggressively if there is to be any curtailment of the looming epidemic.

This survey, representing patients in a developing country, indicates that a large proportion of patients on LLDs are not reaching the accepted LDL-C goals. While these percentages are not dissimilar to those from other studies in Europe, they are below what is currently achieved in North America. The percent of patients at goal in North America has risen over the course of six years, from the NHANES survey of 1999/2000 and the follow up conducted in 2005/2006, indicating that increased awareness and education can result in a greater percentage of patients reaching LDL-C goals, despite the targets becoming more stringent.

The current study indicates both ethnic and gender variances in cardiovascular risk-factor distribution and control in South Africa. Smoking was less prevalent in those of African ancestry and very few black females were smokers. In general black subjects used fewer cigarettes than their Caucasian counterparts (possibly due to economic constraints). The South African Heart of Soweto study confirms that African patients have the lowest smoking prevalence, with patients of mixed ancestry twice as likely, and Caucasian patients three-fold more likely to be current smokers.17 In the current study of patient on LLDs, patients of mixed ancestry had the highest prevalence of smoking among both males and females, followed by Asian males.

The majority of black subjects did not have a family history of premature heart disease, which probably reflects the evolution of the epidemiological transition in an urbanising population, compared to Caucasians, Asians and patients of mixed ethnicity. The Heart of Soweto study also noted that African patients were least likely to be diagnosed with CAD, and showed similar data to CEPHEUS SA for the Asian patients, who had the highest prevalence of a family history of vascular disease.

Control of DM was particularly poor in both male and female African subjects compared with their ethnic counterparts. This may have been due to differences in access to guideline-based management protocols. Despite a high prevalence of the metabolic syndrome in African females, with poor control of DM, their TG levels were the lowest of all subjects – male and female.

The Heart of Soweto study noted that patients of African descent had significantly lower total cholesterol (TC), LDL-C and triglyceride (TG) levels compared to other ethnicities.^17^ These patients were not receiving LLDs. In CEPHEUS SA, in the African-ancestry group, the TGs were not elevated despite a high prevalence of DM with poor control. This would lend credence to the finding that this population group may inherently have low TG levels, and the influence of DM on TGs may be muted.

The Heart of Soweto study confirmed a high prevalence of obesity in patients of African ancestry (43% of the patients having a body mass index greater than 30 kg/m^2^). This substantial burden of obesity among African subjects points to an elevated risk for the future development of DM. Given that DM in this group is poorly controlled, the ameliorating influence of lipid-lowering therapy on future cardiovascular risk could potentially be undermined, or at the very least minimised.

The number of African-ancestry patients who had CAD was low in proportion to the other ethnic groups; however these percentages reflect a change in the prevalence of a disease that was previously considered to be rare in this population. Other studies from South Africa have indicated a prevalence of CAD of less than 10% in the African population.^18^ The prevalence of CAD in African subjects receiving LLDs in the current study was 15.9%.

The INTERHEART Africa study noted that patients of African ancestry presented with myocardial infarction a mean of 3.8 years earlier than patients from the overall INTERHEART study, and also found no inter-ethnic or gender differences.^1^,^19^ Although data for the INTERHEART Africa study were drawn from patients from sub-Saharan countries, more than 80% of subjects were from South Africa, indicating that the data may be comparable to the current CEPHEUS SA study.

## Limitations

This study had the same limitations that apply to many surveys that differ fundamentally from formal prospective studies. The study population was drawn from those already on LLDs and cannot be extrapolated to the general population. Although attempts were made to sample patients from as wide a spectrum as possible, potential selection bias may still have occurred. All centres were located in urban areas, and the applicability to patients of rural origin cannot be assumed.

The public sector provides healthcare to about 80% of the South African population but it made up only about one-third of the sample. Similarly, the study population does not strictly reflect the ratios of the different ethnic groups residing in South Africa. However all previous studies on lipid-lowering therapy in South Africa were predominantly Caucasian based. Private-sector patients were recruited from a wide variety of both specialist and non-specialist practices. The public-sector patients were predominantly recruited from tertiary-care lipid and diabetes clinics. The majority of African patients came from the public sector.

Several private-sector centres had practitioners who dealt predominantly with patients with DM, and this could have further swayed the emphasis of the results on the diabetic cohort. DM is often associated with an increase in body mass index and other anthropometric measures of obesity, and data from a cohort with a high prevalence of DM may therefore not be reflective of the general population.

As the veracity of the patient questionnaires was not tested, the validity of the CVD history may have been inaccurate. Measured clinical parameters (such as blood pressure) were from a single visit and methods of measurement were not standardised or checked, and therefore inaccuracies could have arisen. Causal correlations were not established, and relationships should therefore be interpreted with caution.

## Conclusion

Management of lipid-lowering treatment in South Africa is sub-optimal, and in general lags behind control achieved in the more developed nations. Furthermore, other cardiovascular risk factors are not receiving due attention and their prevalence in this population remains high. For any serious impact to be made on the looming epidemic of cardiovascular disease in the underdeveloped world, more attention needs to be focused on more aggressive treatment of dyslipidaemia as well as the other cardiovascular risk factors and, in particular, diabetes mellitus and obesity.
